# Motion-Blur-Free High-Speed Video Shooting Using a Resonant Mirror

**DOI:** 10.3390/s17112483

**Published:** 2017-10-29

**Authors:** Michiaki Inoue, Qingyi Gu, Mingjun Jiang, Takeshi Takaki, Idaku Ishii, Kenji Tajima

**Affiliations:** 1 Department of System Cybernetics, Hiroshima University, 1-4-1 Kagamiyama, Higashi-Hiroshima, Hiroshima 739-8527, Japan; inoue@robotics.hiroshima-u.ac.jp (M.I.); m-jiang@robotics.hiroshima-u.ac.jp (M.J.); takaki@hiroshima-u.ac.jp (T.T.); 2 Institute of Automation, Chinese Academy of Sciences, No. 95 Zhongguancun East Road, Haidian District, Beijing 100190, China; qingyi.gu@ia.ac.cn; 3 Photron Ltd., Kanda Jinbo-cho 1-105, Chiyoda-Ku, Tokyo 101-0051, Japan; tajima@photron.co.jp

**Keywords:** high-speed vision, frame-by-frame intermittent tracking, fast-moving-object inspection, image stabilization

## Abstract

This study proposes a novel concept of actuator-driven frame-by-frame intermittent tracking for motion-blur-free video shooting of fast-moving objects. The camera frame and shutter timings are controlled for motion blur reduction in synchronization with a free-vibration-type actuator vibrating with a large amplitude at hundreds of hertz so that motion blur can be significantly reduced in free-viewpoint high-frame-rate video shooting for fast-moving objects by deriving the maximum performance of the actuator. We develop a prototype of a motion-blur-free video shooting system by implementing our frame-by-frame intermittent tracking algorithm on a high-speed video camera system with a resonant mirror vibrating at 750 Hz. It can capture 1024 × 1024 images of fast-moving objects at 750 fps with an exposure time of 0.33 ms without motion blur. Several experimental results for fast-moving objects verify that our proposed method can reduce image degradation from motion blur without decreasing the camera exposure time.

## 1. Introduction

High-speed cameras are widely used in high-frame-rate (HFR) video shooting for fast-moving scenes in various applications such as factory inspection, biomedicine, multimedia and civil engineering. In HFR video shooting, the camera’s exposure time should be lowered as the apparent speeds of the target scenes increase to reduce motion blur. Image degradation due to motion blur is affected by the camera’s exposure time, as well as the target speeds. However, the HFR images captured with a lower exposure time become too dark for observation when fast-moving scenes are shot in low light because of insufficient intensity of the light projected on the image sensor. When video shooting fast-moving scenes with high magnification, such as precise product inspection on a conveyor line, road surface and tunnel wall inspection from a fast-moving car and flowing cells in microscopic fields, the trade-off between brightness and motion blur in video shooting is distinctly aggravated. This is because the light intensity projected on the image sensor is lowered and the apparent speed increases with increasing magnification enabled for precise observation.

In order to reduce image degradation due to motion blur when observing moving scenes, many motion deblurring methods [1,2] have been proposed to restore the blurred images by deconvolution using the estimated blur kernels that express the degrees and distributions of motion blur in the images. Blind deconvolution methodologies were used to estimate the blur kernels from a single image using parametric models for maximum a posteriori estimation [3,4,5]. In addition, various types of single-image motion deblurring methods have been proposed for correct prediction of true edges using filters [6,7,8], multi-scale coarse-to-fine approaches [9,10] and reduction of ill-posed image priors in the deblurred images such as normalized sparsity priors [11], color priors [12], patch priors [13], dark channel priors [14] and smoothness priors [15]. Multi-image motion deblurring methods have been used to accurately estimate the blur kernels from multiple images such as super-resolution for consecutively captured images [16,17,18], a high-resolution still camera with a video camera [19,20] and image deblurring with blurred image pairs [21,22]. Considering a camera motion model such as a perspective motion model [23] and simplified three-DOF models [24,25], several studies have reported motion deblurring systems by estimating the camera’s egomotion with gyro sensors and accelerometers [26] or the camera’s geometric location [27]. Most of these motion deblurring methods dealt with image restoration of input images degraded due to motion blur, and they did not consider the acquisition of non-blurred input images. There were limitations to the extent to which the images could be improved, and it was difficult to completely eliminate motion blur in the input images when significant changes with large displacement occurred in the images.

In this study, we propose a novel concept for motion-blur-free video shooting that can capture non-blurred images of fast-moving objects without lowering the camera’s exposure time. Building on the camera-driven frame-by-frame intermittent tracking method [28] in which the actuators are simultaneously controlled for tracking in synchronization with the camera’s frame timings, we extend the method to the actuator-driven frame-by-frame intermittent tracking method so that the camera’s frame timings are controlled for motion-blur-free video shooting in synchronization with the large amplitude vibration of a free-vibration-type actuator such as a resonant mirror vibrating at a high frequency corresponding to its natural frequency. The proposed method can derive the maximum performance of the free-vibration-type actuator that enables motion-blur-free shooting of faster-moving objects at a higher frame rate. The remainder of this paper is organized as follows. Related works on image stabilization for blur reduction, high-speed vision and camera-driven frame-by-frame intermittent tracking method are presented in greater detail in Section 2. In Section 3, we propose the actuator-driven frame-by-frame intermittent tracking method and describe how to determine the parameters in frame-by-frame intermittent tracking. Section 4 provides an outline of the configuration of our motion-blur-free video shooting system with a resonant mirror vibrating at 750 Hz and describes the algorithm implemented for motion-blur-free video shooting of 1024 × 1024 images at 750 fps. The parameters are verified in the preliminary experiments described in Section 5. In Section 6, the effectiveness of the method is verified by demonstrating the results of HFR video shooting experiments performed for fast-moving scenes.

## 2. Related Works

### 2.1. Image Stabilization

To reduce undesirable motion resulting from shaking or jiggling of the camera, a large number of image stabilization techniques has been developed. These techniques can be categorized into: (1) optical image stabilization (OIS) and (2) digital image stabilization (DIS). The lens-shift OIS systems have been designed to shift their optical path using optomechatronic devices such as shift-mechanisms for lens barrels [29,30], a fluidic prism [31], a three-DOF lens platform with magnetic actuation [32] and a deformable mirror [33]. For small systems such as mobile phones, the sensor-shift OIS systems have been compactly designed to shift their image sensors using voice coil actuators [34,35,36,37,38,39]. Many types of multi-DOF gimbal control systems [40,41,42,43,44] have been also used in OIS systems in handheld shooting and drone-based aerial videography with ready-made commercial digital cameras. These OIS systems can stabilize input images for reducing motion blur resulting from camera shake by controlling the optical path with the camera’s internal sensors such as gyro sensors. However, these systems are not suitable for shooting blur-free images of fast-moving scenes when the camera is fixed. This is because the internal sensors cannot detect any apparent motion in the captured images. DIS systems can stabilize input images by compensating the residual fluctuation motion using an image processing technique that estimates the local motion vectors such as block matching [45,46,47], bit-plane matching [48,49], feature point matching [50,51,52,53,54,55] and optical flow estimation [56,57,58,59]. Most of these DIS systems do not need any additional mechanical or optical device, and this feature makes them suitable for low-cost electronics. However, these systems are not suitable for capturing non-blurred input images because they cannot address existing motion blur in the captured images. Since its origination in [60], many high-speed photography methods and systems with strobe lights [61,62,63,64] have been developed. They can shoot videos of fast-moving objects without motion blur with very short strobe pulses, whereas they cannot shoot videos of fast-moving objects at distant places under daylight conditions because ambient light becomes dominant.

### 2.2. High-Speed Vision

In order to track fast-moving objects with visual feedback, many real-time high-speed vision systems operating at 1000 fps or more have been developed [65,66,67,68]. Various types of image processing algorithms such as optical-flow [69], camshift tracking [70], multi-object tracking [71], feature point tracking [72] and face-tracking [73] have been implemented for HFR visual tracking accelerated by field-programmable gate arrays (FPGAs) and graphic processing units (GPUs) on high-speed vision systems. The effectiveness of high-speed vision has been demonstrated in tracking applications such as robot manipulation [74,75,76,77], multicopter tracking [78,79], microscopic cell analysis [80,81,82,83] and vibration analysis [84]. The tracking performances of most of these tracking systems are limited by the time delay of dozens of frames for convergence in tracking control, because the responsive speed of the actuator is much slower than those in the accelerated video capturing and processing in high-speed vision systems. Recently, the 1-ms auto pan-tilt system [85] using galvano-mirrors with accelerated pan-tilt actuators has achieved dynamic image control for ultrafast tracking of moving objects, and such galvano-mirror-based active vision systems can function as virtual multiple tracking cameras that can observe hundreds of different views in a second [86]. By tracking an object to be observed in the center of the camera view with visual feedback, such high-speed tracking systems can reduce motion blur without decreasing their exposure time because the apparent motion of the object to be observed can be canceled in the camera view when the tracking control works correctly. However, motion-blur-free video shooting in such systems is limited to a single target object because the viewpoints cannot be freely changed for observing other objects when the target object is tracked in the camera view.

### 2.3. Camera-Driven Frame-By-Frame Intermittent Tracking

**Figure 1:**
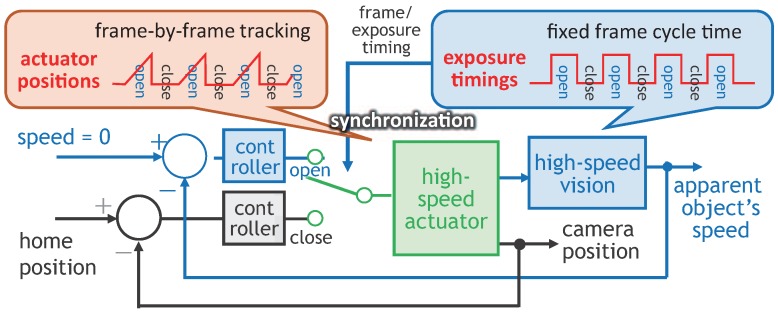
Control scheme in camera-driven frame-by-frame intermittent tracking.

For viewpoint-free video shooting of fast-moving objects without increasing motion blur, Inoue et al. had proposed a frame-by-frame intermittent tracking method [87] that can reduce motion blur by alternating different gaze control methods on an ultrafast active vision system from tracking control to back-to-home control at every frame. In synchronization with the camera shutter timings, the tracking control is executed to maintain the apparent velocity of the object to be observed on the image sensor at zero for motion blur reduction when the camera shutter is open. The back-to-home control is executed to reset the optical path of the camera to its home position without degrading the image quality when the camera shutter is closed because the image sensor is blind to any apparent motion according to no incident light. Figure 1 shows the control scheme of the camera-driven frame-by-frame intermittent tracking, in which the actuator for the alternative gaze control is synchronized with the fixed frame cycles of the camera.

Based on the concept of the camera-driven frame-by-frame intermittent tracking, high-speed mirror-drive tracking systems using high-frequency response actuators such as piezo-mirrors [87,88] and galvano-mirrors [89,90] have been reported for motion-blur-free video shooting of fast-moving objects at hundreds of frames per second. In [87], a mirror-drive two-degrees-of-freedom (DOF) piezo-actuator-based tracking system that can capture 512 × 512 images of fast-moving objects at 125 fps with an exposure time of 4 ms without motion blur was proposed. Two piezo-mirrors with 30 mm × 30 mm mirror surfaces were used, and their movable ranges in pan and tilt directions were very narrow; 0.17 and 0.14 degrees, respectively. Additionally, the trajectory of the piezo-mirror had considerable ripples at its natural frequency of approximately 800 Hz once the motor command was provided to the piezo-mirror. Further, it took 4 ms or more for decaying the ripples. This system could not perform frame-by-frame intermittent tracking at a frame rate larger than 125 fps, and the maximum angular speeds for the pan and tilt angles were limited to 67.1 and 49.7 ${}^{\circ }$ /s, respectively. Thus, there remained the following constraints of a high-frequency response actuator in the camera-driven frame-by-frame intermittent tracking:

(1) Limited movable range 

A high-frequency response actuator has to perform a trade-off between its frequency response and movable range. The amplitude of the repetitive motion at a high frequency is limited because the movable range of a high-frequency response actuator gets narrower as its mechanical time constant gets smaller. In frame-by-frame intermittent tracking with the camera exposure time, the high-frequency response actuator should continuously track a target object whenever the camera shutter is open. However, the motion blur cannot be completely eliminated when the distance of the object during the time the camera shutter is open is larger than the movable range of the actuator. The admissible speed of the target object is limited in motion-blur-free video shooting with a large camera exposure time.

(2) Limited controllability in the high-frequency range 

A high-frequency response actuator requires a certain time to attenuate its ringing response with resonant vibration, because it achieves its high-frequency drive with a low damping ratio by reducing its viscosity such as friction. The trajectory of a high-frequency response actuator should be linearly controlled whenever the camera shutter is open so as to cancel the apparent speed of the target object assuming that it moves at a fixed speed as long as the camera shutter is open. However, it is difficult to completely eliminate ripples in the actuator’s trajectory in the frame-by-frame intermittent tracking at hundreds of hertz or more because the frame interval is not larger than its damping time for resonant vibration, and motion blurs are still retained in the images.

## 3. Actuator-Driven Frame-By-Frame Intermittent Tracking

### 3.1. Concept

According to the constraints stated in the previous section, the camera-driven frame-by-frame intermittent tracking method cannot always derive the maximum performance of a high-frequency response actuator, and the frame rate of a high-speed vision system should be lowered so as to maintain the linear trajectory of the actuator during the time the camera shutter is open. The very flexible controllability of the high-speed vision system, whose frequency response is much higher than that of the actuator, was not fully utilized in the frame-by-frame intermittent tracking.

**Figure 2:**
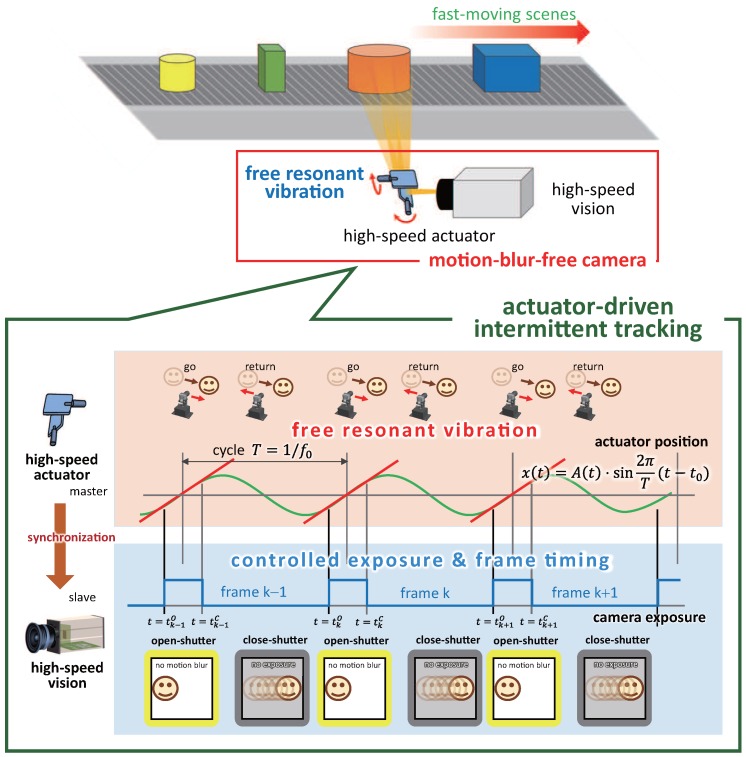
Concept of the actuator-driven frame-by-frame intermittent tracking.

Thus, in this study, we propose an improved frame-by-frame intermittent tracking method that can reduce motion blur in video shooting by controlling the camera shutter timings in synchronization with the resonant vibration of a free-vibration-type actuator such as a resonant mirror. Its high-frequency vibration with a large amplitude enables the ultrafast gaze control to track fast-moving objects during the time the camera shutter is open. Figure 2 shows the concept of our proposed actuator-driven frame-by-frame intermittent tracking method.

When the camera’s viewpoint moves unidirectionally, the viewpoint’s position x(t) at time *t* vibrates at a cycle time of $T=1/f_{0}$ on the following sinusoid trajectory,

(1) $$\begin{array}{c} x\left(t\right)=A\left(t\right)\cdot \sin\frac{2\pi }{T}t, \end{array}$$

where $f_{0}$ is the resonant frequency of the free-vibration-type actuator and A(t) is the amplitude of the vibration at time *t*, assuming x(t)=0 when t=0 . In the actuator-driven tracking approach, the exposure start and end times to capture the image at frame *k*, which are expressed as $t_{k}^{O}$ and $t_{k}^{C}$ , respectively, are controlled so that the camera shutter is open when the viewpoint is located in the highly linear range within the sinusoid trajectory. In parallel with the shutter timing control, the slope of the approximate line to the sinusoid trajectory when the camera shutter is open, which indicates the speed of the camera’s viewpoint, is controlled for motion blur reduction so as to coincide with the apparent speed of the target object on the image sensor. In the frame-by-frame intermittent tracking with a free-vibration-type actuator, the resonant frequency, which is a fixed value peculiar to the actuator, is not controllable, and the speed of the camera’s viewpoint can be controlled with the amplitude of the vibration, as well as the exposure start and end times, which determine the time range for the linear approximation to the sinusoid trajectory.

**Figure 3:**
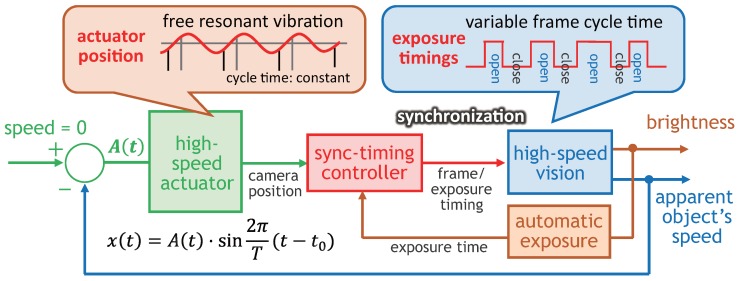
Control scheme in actuator-driven frame-by-frame intermittent tracking.

Figure 3 shows the control scheme of our actuator-driven tracking approach. Compared to the performance-limited mechanical actuator control in the camera-driven tracking approach, the actuator-driven tracking approach can derive the maximum mechanical performance of a free-vibration-type actuator enables motion-blur-free video shooting of faster moving objects at a higher frame rate, whereas a free-vibration-type actuator is plagued by the following limitations:

(1) Unresponsive amplitude control in resonant vibration 

A free-vibration-type actuator tends to move on a periodic trajectory with a certain hysteresis caused by friction, and it is largely deviated from the ideal sinusoid trajectory in the case of the resonant vibration with a small amplitude. In the actuator-drive tracking approach, such properties may degrade the tracking performance in video shooting a target object whose speed is either very low or varies with time.

(2) Limited time aperture ratio 

In the camera-driven tracking approach, the time aperture ratio, which is the ratio of the frame interval and the exposure time in video shooting, can be programmably determined by designing the target trajectory of the camera’s viewpoint freely, whereas the high-frequency response actuator cannot move on the target trajectory with a large amplitude, due to its limited movable range and speed. On the other hand, the time aperture ratio in the actuator-driven tracking approach is limited due to the sinusoid trajectory with resonant vibration. This is because the camera shutter timings are automatically determined so as to guarantee the linear motion of the camera’s viewpoint when the camera shutter is open, whereas the percentage of the linear range on the sinusoid trajectory decreases as the exposure time increases.

### 3.2. Camera Shutter Timings and Vibration Amplitude

In motion-blur-free video shooting with actuator-driven frame-by-frame intermittent tracking, the nonlinear sinusoid trajectory with resonant vibration of a free-vibration-type actuator, which is segmented in the time range when the camera shutter is open, deviates from its approximate straight line more extensively as the camera exposure time increases. For motion-blur-free video shooting without lowering the incident light, it is important to determine a larger camera exposure time with consideration of the permissible deviation error in straight-line approximation, which corresponds to the degree of motion blur. In this subsection, we discuss how to determine parameters for camera shutter timings in actuator-driven frame-by-frame intermittent tracking on the basis of the numerical relationship between the segmented sinusoid trajectory and its approximate straight line.

**Figure 4:**
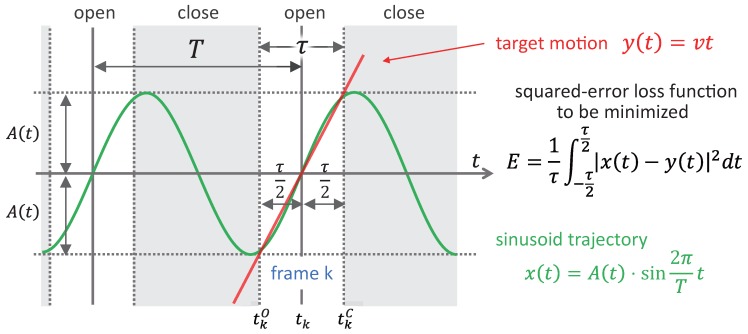
Sinusoid trajectory and its approximate straight line.

As illustrated in Figure 4, the input image is captured at frame *k* with an exposure time τ by opening and closing the camera shutter at times $t_{k}^{O}=t_{k}-\tau /2$ and $t_{k}^{C}=t_{k}+\tau /2$ , respectively. In this study, we assume that the center time of the camera exposure is set to $t_{k}=2n\pi$ (*n*: integer) to synchronize with the sinusoid trajectory x(t)=Asin(2π/T)t so that the slope of a tangent to the sinusoid trajectory is maximum at time $t_{k}$ . To track a target object moving at a speed of *v* in images when the camera shutter is open, we assume that the amplitude *A* of the sinusoid trajectory is so controlled that the straight line y(t)=vt approximates the segmented sinusoid trajectory in the range of time $t_{k}^{O}$ to time $t_{k}^{C}$ . Here, we assume that the open and close times for camera exposure are $t_{k}^{O}=-\tau /2$ and $t_{k}^{C}=\tau /2$ , respectively, by setting the center time to $t_{k}=0$ for simplification, and the *y*-intercept of the approximate line is zero because the segmented sinusoid trajectory in the range of time $t_{k}^{O}$ to time $t_{k}^{C}$ is symmetric about the center time $t_{k}$ . To estimate the amplitude *A*, we consider a minimization problem for the following squared-error loss function that can evaluate the deviation of the segmented sinusoid trajectory from the straight line where the target object moves,

(2) $$\begin{array}{ccc} E(A) & = & \frac{1}{t_{k}^{C}-t_{k}^{O}}\int_{t_{k}^{O}}^{t_{k}^{C}}{\left|x(t)-y(t)\right|}^{2}dt=\int_{t_{k}^{O}}^{t_{k}^{C}}{\left|A\sin\frac{2\pi }{T}t-vt\right|}^{2}dt,\\ & = & \frac{1}{t_{k}^{C}-t_{k}^{O}}\left(A^{2}\int_{t_{k}^{O}}^{t_{k}^{C}}\sin^{2}\frac{2\pi }{T}tdt-2Av\int_{t_{k}^{O}}^{t_{k}^{C}}t\sin\frac{2\pi }{T}tdt+v^{2}\int_{t_{k}^{O}}^{t_{k}^{C}}t^{2}dt\right),\\ & = & A^{2}\cdot \left(\frac{1}{2}-\frac{T}{4\pi \tau }\sin\frac{2\pi \tau }{T}\right)-2Av\cdot \left(-\frac{T}{2\pi }\cos\frac{\pi \tau }{T}+\frac{T^{2}}{2\pi^{2}\tau }\sin\frac{\pi \tau }{T}\right)+v^{2}\cdot \frac{\tau^{2}}{12}. \end{array}$$

**Figure 5:**
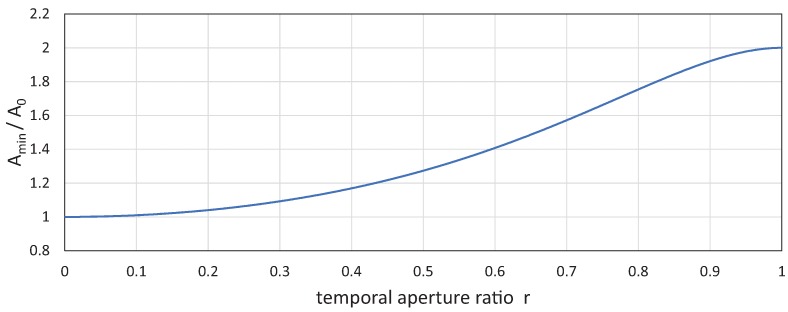
Relationship between temporal aperture ratio *r* and the amplitude ratio of $A_{min}$ to $A_{0}$ .

Solving the following equation such that the partial derivative of E(A) with respect to *A* is zero,

(3) $$\begin{array}{c} \frac{\partial E}{\partial A}=2A\cdot \left(\frac{1}{2}-\frac{T}{4\pi \tau }\sin\frac{2\pi \tau }{T}\right)-2v\cdot \left(-\frac{T}{2\pi }\cos\frac{\pi \tau }{T}+\frac{T^{2}}{2\pi^{2}\tau }\sin\frac{\pi \tau }{T}\right)=0, \end{array}$$

the amplitude $A_{min}$ can be derived as follows:

(4) $$\begin{array}{c} A_{min}=\frac{T^{2}\left(\sin\frac{\pi \tau }{T}-\frac{\pi \tau }{T}\cos\frac{\pi \tau }{T}\right)}{\pi^{2}\tau \left(1-\frac{T}{2\pi \tau }\sin\frac{2\pi \tau }{T}\right)}\cdot v=\frac{T(\sin\pi r-\pi r\cos\pi r)}{\pi^{2}r\left(1-\frac{\sin2\pi r}{2\pi r}\right)}\cdot v, \end{array}$$

where r=τ/T is the temporal aperture ratio that indicates the ratio of the exposure time τ to the cycle time *T* of the sinusoid trajectory. Figure 5 shows the relationship between the temporal aperture ratio *r* and the amplitude ratio of $A_{min}$ to $A_{0}$ ; $A_{0}$ is the slope of the tangent line of the sinusoid trajectory at time $t_{k}$ when the exposure time τ approaches zero as follows:

(5) $$\begin{array}{c} A_{0}=\lim_{\tau \to 0}A_{\min}=\frac{T}{2\pi }v. \end{array}$$

Thus, the minimum value $E_{min}$ of the squared-error loss function is obtained as follows:

(6)Emin=minAE(A)=E(Amin)=τ212−T3π3τ(sinπτT−πτTcosπτT)22πτT−sin2πτTv2,(7)=T2rr312−1π3(sinπr−πrcosπr)22πr−sin2πrv2.

Without actuator-driven frame-by-frame intermittent tracking, the squared-error loss $E_{NT}$ in the range of time $t_{k}^{O}$ to time $t_{k}^{C}$ when observing a target object moving at speed *v* can be described as the value of the loss function when the amplitude of the sinusoid trajectory is A=0 , corresponding to no camera motion for tracking, as follows:

(8) $$\begin{array}{c} E_{NT}=E\left(A=0\right)=\frac{1}{t_{k}^{C}-t_{k}^{O}}\int_{t_{k}^{O}}^{t_{k}^{C}}{\left|vt\right|}^{2}dt=\frac{\tau^{2}}{12}\cdot v^{2}=\frac{T^{2}r^{2}}{12}\cdot v^{2}. \end{array}$$

Considering the roots of the squared-error losses of $E_{min}$ and $E_{NT}$ , the relative error ratio ε is defined as follows:

(9) $$\begin{array}{c} \varepsilon =\frac{\sqrt{E_{min}}}{\sqrt{E_{NT}}}=\sqrt{1-\frac{12}{\pi^{3}r^{3}}\frac{{\left(\sin\pi r-\pi r\cos\pi r\right)}^{2}}{2\pi r-\sin2\pi r}}, \end{array}$$

where ε indicates the degree of motion blur reduction in video shooting with frame-by-frame intermittent tracking, compared with the deviation error in video shooting without tracking; ε=f(r) is a monotonically increasing function of the temporal aperture ratio *r*, and motion blur is largely canceled when ε approaches zero. Thus, the temporal aperture ratio *r* can be expressed as a monotonically increasing function of the relative error ratio ε , which is independent of the cycle time *T* of the sinusoid trajectory and the target speed *v*, as follows:

(10) $$\begin{array}{c} r=f^{-1}\left(\varepsilon \right). \end{array}$$

**Figure 6:**
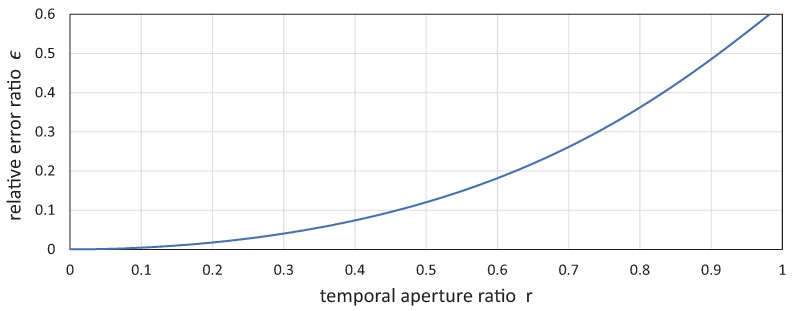
Relationship between temporal aperture ratio *r* and relative error ratio ε .

Figure 6 shows the relationship between the temporal aperture ratio *r* and the relative error ratio ε . Using the relationship between *r* and ε in Figure 6 as a look-up table, the camera shutter timings can be automatically determined in actuator-driven frame-by-frame intermittent tracking when the permissible degree of motion blur is initially given. For example, the relative error ratio ε is permissible up to 1%, 5% and 10%, respectively, and the upper-limit values of the allowable temporal opening ratios are r(0.01)=0.151 , r(0.05)=0.333 , and r(0.1)=0.460 , respectively. Especially when the exposure time is constant, the open and close times for camera exposure can be determined independently from the time-varying amplitude *A* of the sinusoid trajectory, which is controlled so as to cancel the apparent speed of the target object when the camera exposure is open, and these signals are generated in synchronization with the external synchronization signal from a free-vibration-type actuator.

## 4. Motion-Blur-Free HFR Video Shooting System

**Figure 7:**
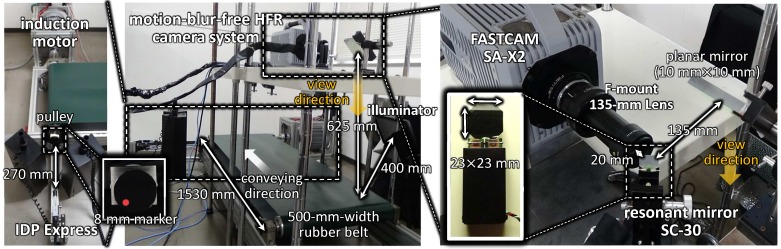
Overview of the test-bed system for motion-blur-free video shooting.

In order to verify the effectiveness of actuator-driven frame-by-frame intermittent tracking, we developed a test-bed system for motion-blur-free HFR video shooting. Figure 7 shows the overview of the test-bed system. The test-bed system consists of (1) a motion-blur-free HFR camera system with a resonant mirror and (2) a high-speed belt-conveyor system that can convey target objects to be observed at various speeds.

The motion-blur-free HFR camera system consists of a high-speed video camera (FASTCAM SA-X2, Photron, Tokyo, Japan) with an F-mount 135-mm lens (Rodagon 135 mm F5.6, Qioptiq Photonics, Hamble-le-Rice, UK), a resonant mirror (SC-30, Electro-Optical Products, Ridgewood, NY, USA), a function generator (AFG1022, Tektronix, Beaverton, OR, USA) and a personal computer (PC) with an ASUSTeK P6T7 WS Supercomputer main board, Intel Core i7 960 3.20-GHz CPU, 6-GB memory, Windows 7 Professional 32-bit OS and a D/A board (PEX-340416, Interface, Hiroshima, Japan). FASTCAM SA-X2 has a 12-bit 1024 × 1024 CMOS image sensor, the sensor size and pixel size of which are 20.48 mm × 20.48 mm and 20 μ m × 20 μ m, respectively, and it can capture and record 1024 × 1024 images at 12,500 fps in inner memories with a 256-parallel analog output at a 65-MHz clock. The camera parameters such as exposure time can be set through a Gb Ethernet from external systems, whereas the shutter timings to open the camera exposure can be directly determined by an external trigger signal. SC-30 is a resonant mirror system that can vibrate a 23-mm × 23-mm-size glass mirror in the pan direction at its resonant frequency of 750 Hz. The amplitude of its sinusoid trajectory can be controlled in the range of 0.0025 to 0.5 ${}^{\circ }$ by providing a voltage command in the range of 0 to 5 V to its automatic gain control driver; the voltage command was outputted from the D/A board mounted on the PC. A transistor-transistor-logic (TTL) signal is externally outputted at the cycle time of its resonant vibration, and it was used to determine the shutter timings to open the camera exposure at 750 Hz. The function generator AFG1022 was used to adjust the delay time in the external TTL signal so as to synchronize the center time of the open exposure with the vibration center of the sinusoid trajectory in this study. The PC was mainly used to control the vibration amplitude of the resonant mirror system for motion-blur-free video shooting.

The high-speed belt-conveyor system was installed 625 mm below a planar mirror of 100 mm × 100 mm size; the planar mirror was located 135 mm on the right side of the resonant mirror to change the direction of the camera view to the vertical direction for target objects horizontally-moving on the belt-conveyor system; they were observed under the lighting with an LED illuminator (VLP-10500XP, LPL, Saitama, Japan), which was installed 400 mm diagonally upward from the belt-conveyor system. On the belt-conveyor system, target objects attached to a 500-mm-width rubber belt can move forward with rotations of 80 mm-diameter pulleys, one of which was the drive pulley powered with a three-phase induction motor (SF-PRV-3.7KW-4P-200V, Mitsubishi Electric, Tokyo, Japan). The length between pulleys was set to 1.5 m. The induction motor was controlled by an inverter (WJ200-037LF, Hitachi Industrial Equipment Systems, Tokyo, Japan), and the conveying speed of the belt-conveyor system can be set in the range of 0 to 7.55 m/s by providing a voltage command in the range of 0 to 10 V to the inverter. The rotation speed of the induction motor was measured by a high-speed vision system IDP Express [67]; the rotation speed was computed by extracting the position of an 8 mm-diameter marker attached on a rotational axis with real-time video processing of 512 × 512 images at 2000 fps on IDP Express. Thus, the conveying speed of the belt-conveyor system can be simultaneously estimated for motion-blur-free video shooting at 2000 Hz on the PC, on which IDP Express was mounted for controlling the vibration amplitude of the resonant mirror.

With this test-bed system, 1024 × 1024 input images were captured at 750 fps, with a frame interval of 1.333 ms and the exposure time of 0.33 ms, respectively, in synchronization with the external trigger signal from the resonant mirror, which was dependent on its resonant frequency. The intensity of incident light on the image sensor was not different from that while shooting a video without a resonant mirror. We confirmed that only 2.2% of the intensity of incident light was lost owing to the 23 mm × 23 mm mirror in the presented setup, compared with that in video shooting without a resonant mirror. The temporal aperture ratio in frame-by-frame intermittent tracking was r=0.25 , and the relative error ratio ε=0.028 ; this corresponded to the relationship between *r* and ε in Figure 6. A target object on the belt plane was apparently distant R= 760 mm from the center of the resonant mirror. Considering the twice of the mirror angle of the resonant mirror, the sinusoid trajectory of the mirror angle $\theta \left(t\right)=A_{\theta }\sin2\pi t/T$ was projected on the belt plane as:

(11) $$\begin{array}{c} x\left(t\right)=2A_{\theta }R\sin\frac{2\pi }{T}t, \end{array}$$

where it is assumed that θ(t) is small. From Equation (4), the vibration amplitude $A_{\theta }$ of the resonant mirror to minimize the squared-error loss function described in Equation (3) can be expressed as a function of the speed *v* of a target object by substituting T= 1.333 ms, r= 0.25 and R= 760 mm as follows:

(12) $$\begin{array}{c} A_{\theta }=\frac{1}{2R}\frac{T(\sin\pi r-\pi r\cos\pi r)}{\pi^{2}r\left(1-\frac{\sin2\pi r}{2\pi r}\right)}\times v=c_{min}\times v, \end{array}$$

where $c_{min}=$ 1.485 $\times \;10^{-4}$ (rad·s/m) = 8.506 $\times \;10^{-3}$ ( ${}^{\circ }$ ·s/m). The vibration amplitude of the resonant mirror was controlled with sensor feedback so that the apparent scan speed with the resonant mirror on target objects on the belt was always matched with the conveying speed measured by the high-speed vision system IDP Express. An image region of 1024 × 1024 pixels corresponded to the 104 mm × 104 mm area on the belt of the belt-conveyor system, and 0.10 mm corresponded to one pixel. The maximum permissible speed for target objects to be observed that can guarantee the efficiency of frame-by-frame intermittent tracking was determined theoretically by the ratio of 0.5 deg to 0.33 ms, which were the maximum movable angle of the resonant mirror and the duration time of the exposure time, respectively; the maximum angular speed was 2.34 × 10 ${}^{3}$ ${}^{\circ }$ /s considering that the variation of the view angle via the mirror corresponds to twice that of the mirror angle. Thus, the displacement of 95.6 pixels in the *x* direction on the image sensor was permissible during the open exposure of 0.33 ms, and the maximum permissible apparent speed on the image sensor was 2.90 × 10 ${}^{5}$ pixel/s. This value corresponded to the maximum permissible speed of 29.4 m/s for objects to be observed on the belt of the belt-conveyor system, whereas the maximum conveying speed of the belt-conveyor system was 7.55 m/s.

## 5. Preliminary Experiments

### 5.1. Relationship between Drive Voltage and Vibration Amplitude

**Figure 8:**
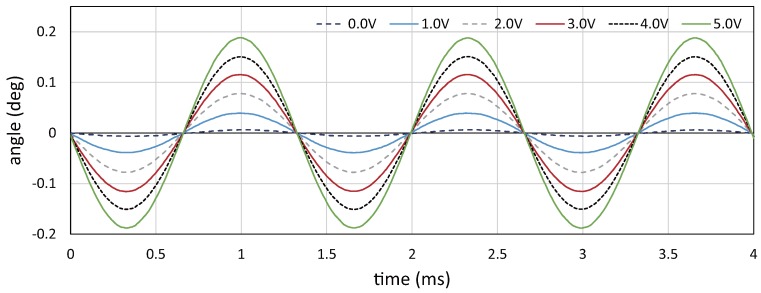
Relationship between drive voltage and angular displacement.

**Figure 9:**
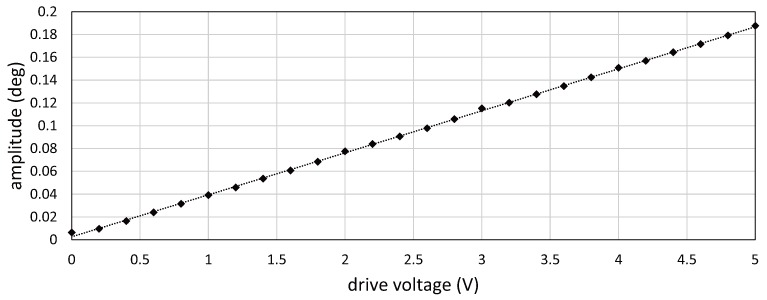
Relationship between drive voltage and vibration amplitude.

**Figure 10:**
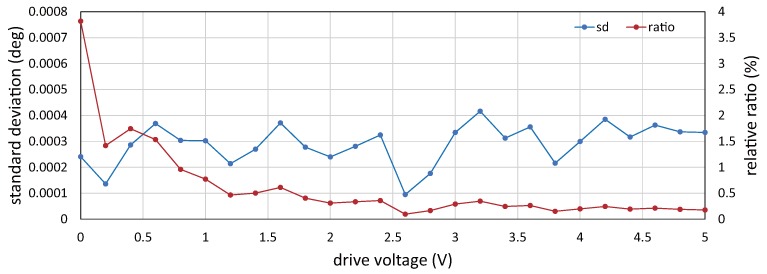
Relationship between drive voltage and the standard deviation of vibration amplitude.

Firstly, we conducted a preliminary experiment to verify the relationship between the drive voltage to the resonant mirror and its angular displacement. To measure the angular displacement, a laser beam spot was redirected by the resonant mirror, and the locations of the beam spots projected on a screen at a distance of 1375 mm from the resonant mirror were extracted offline by capturing an HFR video of 384 × 56 pixels at 100,000 fps with the exposure time of $8.98\times \;10^{-3}$ ms. Figure 8 shows the angular displacement for 4 ms when the drive voltage to the resonant mirror was set to 0.0, 1.0, 2.0, 3.0, 4.0 and 5.0 V. The angular displacement was sinusoidally changed at a frequency of 750 Hz, and its amplitude increased in proportion with the drive voltage. Figure 9 shows the relationship between the drive voltage and the averaged vibration amplitude of the angular displacement for 1 s, corresponding to 750 cycle times of the 750-Hz vibration, when the drive voltage varied in the range of 0.0 to 5.0 V at steps of 0.2 V. The vibration amplitude linearly varied with the amplitude of the drive voltage, whereas there was a slight offset around 0 V; the relationship between the drive voltage *V* (V) and the vibration amplitude *A* ( ${}^{\circ }$ ) can be linearly approximated as A=0.0368V+0.0026 . Figure 10 shows the relationship between the drive voltage and the standard deviation of the vibration amplitude in the duration time of 1 s. In the figure, the relative ratio of the standard deviation to the averaged vibration amplitude was also plotted. When the drive voltage was 5.0 V, the averaged vibration amplitude and its standard deviation were 0.188 and 3.35 × $10^{-4}$ ${}^{\circ }$ , respectively. The standard deviations had similar values around 3 × $10^{-4}$ ${}^{\circ }$ at all the drive voltages, and the relative ratio decreased in proportion to the drive voltage; the relative ratio was 0.18% when the drive voltage was 5 V.

### 5.2. Step Responses of Vibration Amplitude

**Figure 11:**
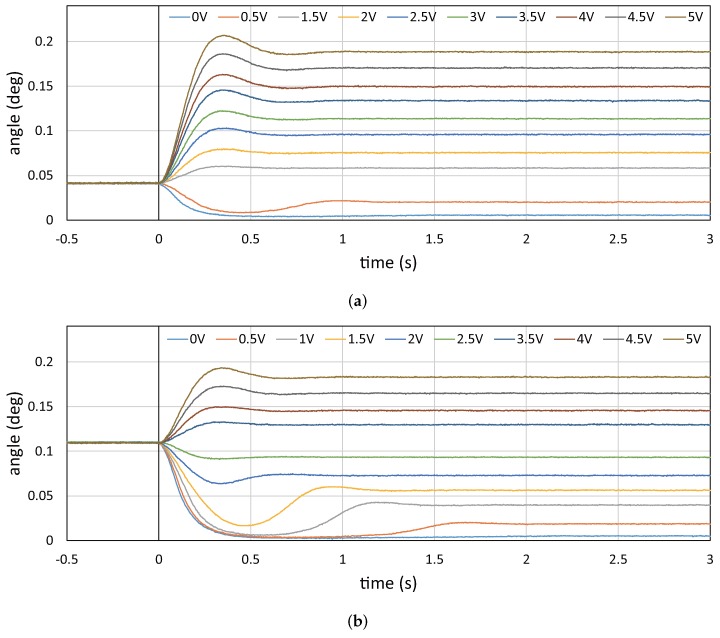
Step response of vibration amplitude: (**a**) 1 V; (**b**) 3 V.

**Figure 12:**
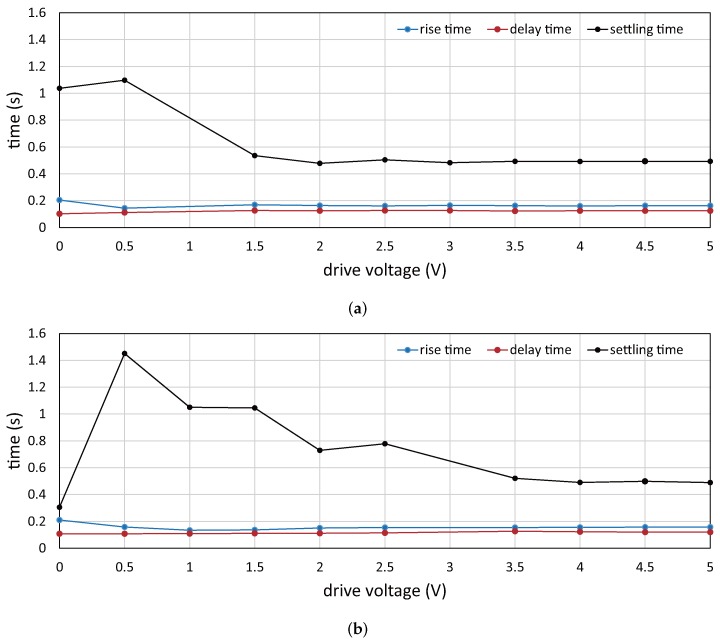
Dynamic response parameters of vibration amplitude: (**a**) 1 V; (**b**) 3 V.

Next, we conducted an experiment to verify the response time of the vibration amplitude of the resonant mirror when a step drive voltage is commanded to the resonant mirror. In a similar environment as that in the previous subsection, the vibration amplitude of the resonant mirror is measured by capturing an HFR video for the laser beam spots projected on a screen; 384 × 265 images were captured for 5 s at 750 Hz with the exposure time of 0.05 ms in synchronization with the timing when the angular displacement of the resonant mirror was at the maximum. Figure 11 shows the step response of the vibration amplitude when the drive voltage of (a) 1 V and (b) 3 V is simultaneously switched to the different target voltage in the range of 0 to 5 V at time t=0 . Figure 12 shows the rise time (from 10 to 90%), the delay time (to 50%) and the settling time (within 5 %) of the vibration amplitude when analyzing the step responses in Figure 11. At all target voltages except 0 V in (a) 1 V and (b) 3 V, the rise times and the delay times had similar values of 0.16 s and 0.12 s, respectively. However, the settling times were much larger than these parameters. There was a distinct tendency of hysteresis that the settling time was around 0.50 s for all cases when the drive voltage increased, whereas it became larger when the drive voltage largely decreased. Comparing with the 750-Hz free vibration of the resonant mirror, the dynamic response of the vibration amplitude is so slow and hysterical that the vibration amplitude cannot be quickly controlled for motion blur reduction when the speed of a target object to be tracked is quickly time-varying, whereas vibration amplitude control functions well in motion-blur-free video shooting for a target object moving at a large, but slightly time-varying speed in many applications such as product inspection on a factory line and road inspection from a moving car.

## 6. Video Shooting Experiments

### 6.1. Video Shooting without Amplitude Control for Circle-Dots Moving at Constant Speeds

**Figure 13:**
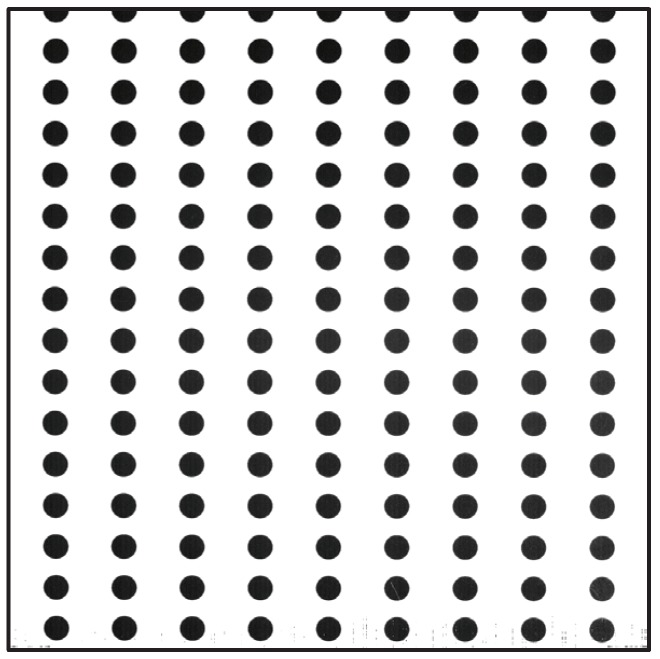
Circle-dot pattern to be observed.

Next, we conducted video shooting experiments for a patterned object attached on the moving belt of the test-bed system to verify the relationship between the speed of a target object and its motion blur when the vibration amplitude of the resonant mirror was set to a constant value. Figure 13 shows the patterned object to be observed;a circle-dot pattern on which 4 mm-diameter circle-dots were black-printed at vertical and horizontal intervals of 11 mm and 7 mm, respectively.

**Figure 14:**
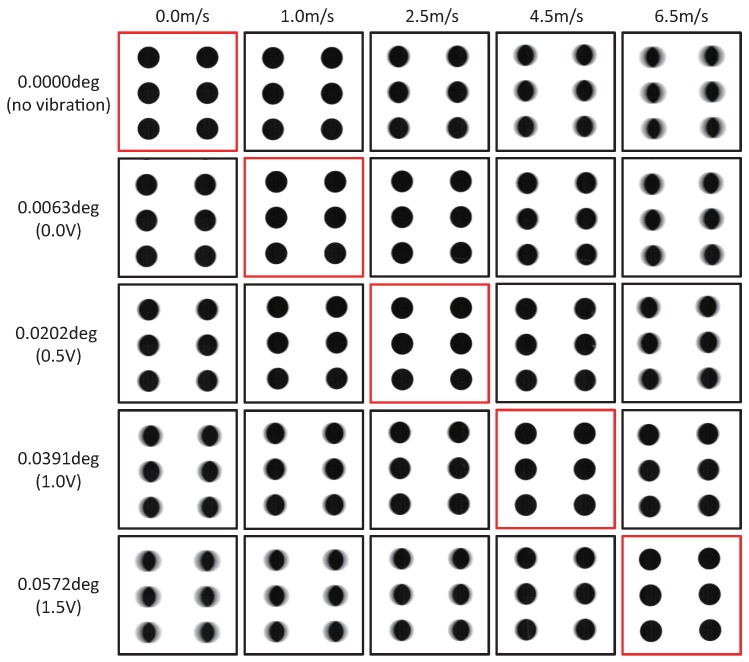
Images of a circle-dot pattern when video shooting without amplitude control.

Figure 14 shows the 215 × 215 images cropped from the 1024 × 1024 input images of the circle-dot pattern moving with the motor command of 0.0, 1.0, 2.5, 4.0 and 6.5 m/s to the conveyor system when the vibration amplitude of the resonant mirror was set to 0.0000 ${}^{\circ }$ , 0.0063 ${}^{\circ }$ , 0.0202 ${}^{\circ }$ , 0.0391 ${}^{\circ }$ and 0.0572 ${}^{\circ }$ ; 0.0000 ${}^{\circ }$ corresponded to no vibration of the resonant mirror, and 0.0063 ${}^{\circ }$ , 0.0202 ${}^{\circ }$ , 0.0391 ${}^{\circ }$ and 0.0572 ${}^{\circ }$ corresponded to the voltage command of 0.0, 0.5, 1.0, 1.5 V to the control driver of the resonant mirror, respectively. The patterns moving with the motor command of 0.0, 1.0, 2.5, 4.5 and 6.5 m/s were captured without motion blur when the vibration amplitude of the resonant mirror was 0.0000 ${}^{\circ }$ , 0.0063 ${}^{\circ }$ , 0.0202 ${}^{\circ }$ , 0.0391 ${}^{\circ }$ and 0.0572 ${}^{\circ }$ , respectively. This tendency corresponded to that the squared-error loss functions when the vibration amplitude of the resonant mirror was 0.0000, 0.0085 ${}^{\circ }$ , 0.0213 ${}^{\circ }$ , 0.0383 ${}^{\circ }$ and 0.0553 ${}^{\circ }$ were minimized in observing a target object moving with the motor command of 0.0, 1.0, 2.5, 4.5 and 6.5 m/s, respectively, according to Equation (12). The image degradation with motion blur in the horizontal direction became larger as the object speed deviated from its desired speed for motion blur reduction, which was determined by the vibration amplitude of the resonant mirror.

For a circle-dot pattern, the blur index $\lambda_{dot}=\lambda_{x}-\lambda_{x}^{0}$ was introduced; $\lambda_{x}$ represents the length of the *x*-axis of the approximated ellipse of the circle dot in the image, and $\lambda_{x}^{0}$ is the value of $\lambda_{x}$ in observing a circle-dot at a fixed location in the case of no vibration of the resonant mirror. The index $\lambda_{dot}$ increases as the motion blur in the horizontal direction becomes larger in the image, and it becomes zero when the circle-dot has no motion. $\lambda_{x}$ was estimated offline by computing its zero-, first- and second-order moment features for the circle-dot region in the 424 × 424 image cropped from the input image; a single circle-dot was located at the center of the cropped image. The circle-dot region was extracted by binarization with a threshold of 2600.

**Figure 15:**
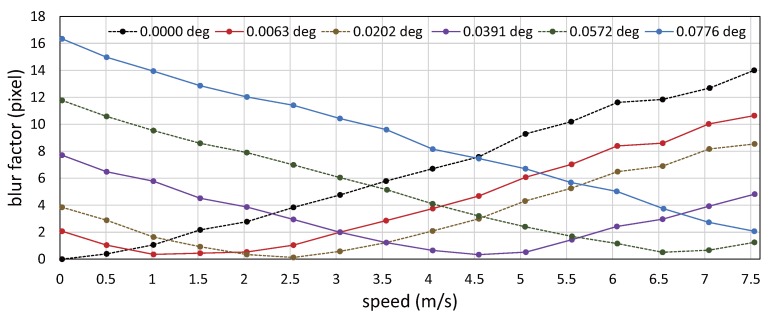
Blur indexes $\lambda_{dot}$ when video shooting without actuator control.

Figure 15 shows the relationship between the speed of a circle-dot and its blur index $\lambda_{dot}$ when the target objects moved with the motor command to the conveyor system in the range of 0.0 to 7.5 m/s at intervals of 0.5 m/s. In the figure, the blur indexes $\lambda_{dot}$ were averaged by those for 25 selected dots in two images, and they were plotted when the vibration amplitude of the resonant mirror was 0.0000 ${}^{\circ }$ , 0.0063 ${}^{\circ }$ , 0.0202 ${}^{\circ }$ , 0.0391 ${}^{\circ }$ , 0.0572 ${}^{\circ }$ and 0.0776 ${}^{\circ }$ . When the vibration amplitude was 0.0000 ${}^{\circ }$ , 0.0063 ${}^{\circ }$ , 0.0202 ${}^{\circ }$ , 0.0391 ${}^{\circ }$ and 0.0572 ${}^{\circ }$ , the blur index $\lambda_{dot}$ had a minimum value when the motor command was 0.0, 1.0, 2.5, 4.5 and 6.5 m/s, respectively; these motor commands were around the desired speeds for motion blur reduction, 0.00, 0.74, 2.38, 4.60 and 6.72 m/s, which were determined by the vibration amplitude 0.0000 ${}^{\circ }$ , 0.0063 ${}^{\circ }$ , 0.0202 ${}^{\circ }$ , 0.0391 ${}^{\circ }$ and 0.0572 ${}^{\circ }$ , respectively. The desired speed for motion blur reduction when the vibration amplitude of the resonant mirror was 0.0776 ${}^{\circ }$ , corresponding to the voltage command of 2.0 V to its control driver, was 9.12 m/s, and it was larger than the maximum conveying speed of 7.55 m/s on the belt-conveyor system. Thus, there were no local maximum/minimum values when the vibration amplitude was 0.0776 ${}^{\circ }$ in Figure 15. These experimental results indicate that we can reduce motion blur in video shooting when the object speed corresponds to the desired speed for motion blur reduction, which is determined by the vibration amplitude of the resonant mirror.

### 6.2. Video Shooting with Amplitude Control for Circle-Dots Moving at Constant Speeds

Next, we conducted video shooting experiments for a fast-moving patterned object when the vibration amplitude of the resonant mirror was controlled in proportion to the object speed on the belt, which was estimated at 2000 fps by IDP Express, so that motion blurs in input images were reduced by frame-by-frame intermittent tracking. The circle-dot pattern identical to that used in Section 6.1 was observed in the experiments.

**Figure 16:**
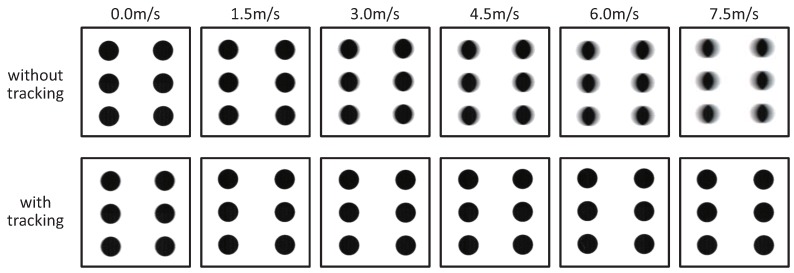
Images of a circle-dot pattern when video shooting with amplitude control.

**Figure 17:**
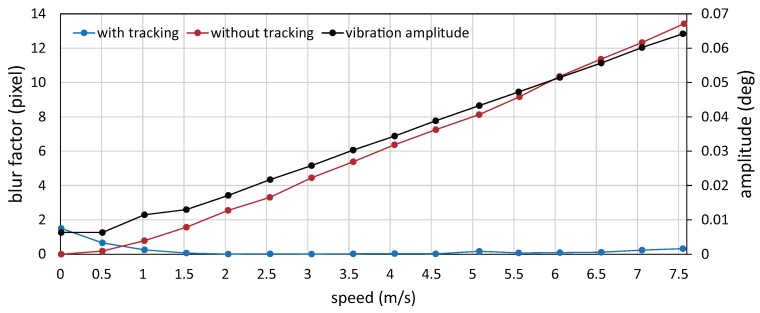
Blur indexes $\lambda_{dot}$ for a circle-dot pattern and the vibration amplitude of resonant mirror when video shooting with actuator control.

Figure 16 shows the 215 × 215 images cropped from the 1024 × 1024 input images of the circle-dot pattern when the patterned objects moved with the motor command to the conveyor system of 0.0, 1.5, 3.0, 4.5, 6.0 and 7.5 m/s. The input images captured when the vibration amplitude of the resonant mirror was controlled with sensor feedback (IT, with tracking) were compared with those captured when no vibration of the resonant mirror (NT, without tracking). As observed in Figure 16, the NT images became increasingly blurred in the horizontal direction as the object speed increased, whereas the IT images were non-blurred for all the speeds. Figure 17 shows the relationship between the speed of a circle-dot and its blur index $\lambda_{dot}$ in video shooting the IT images with amplitude control and the NT images without amplitude control when the target objects moved with the motor command to the conveyor system in the range of 0 to 7.5 m/s at intervals of 0.5 m/s. In the figure, the vibration amplitudes of the resonant mirror in video shooting the IT images with amplitude control were also plotted. The index $\lambda_{dot}$ was computed offline in a similar manner as in the previous section. In Figure 17, the vibration amplitudes of the resonant mirror were controlled for motion blur reduction in proportion to the object speed in video shooting with amplitude control, whereas there remained a slight non-zero offset in the vibration amplitude when the motor command to the conveyor system was 0 m/s. In Figure 17, the blur index $\lambda_{dot}$ for the IT images was remarkably low, comparing with that for the NT images. The blur index $\lambda_{dot}$ for the IT images when the motor command was 0.0, 1.5, 3.0, 4.5, 6.0 and 7.5 m/s was 1.50. 0.07, 0.00, 0.02, 0.09 and 0.33 pixel, respectively, whereas that for the NT images was 0.00, 1.57, 4.45, 7.25, 10.36 and 13.42 pixel, respectively. In the experiments, the object speed was 7.55 m/s or less, which was smaller than the maximum permissible motion-blur-free speed of 29.4 m/s in the horizontal direction, and our actuator-driven frame-by-frame intermittent tracking method remarkably reduced motion blurs of the circle-dot pattern moving at high speed. When the motor command to the conveyor system was 1.0 m/s or less, the blur index $\lambda_{dot}$ in video shooting with actuator control was slightly larger than that in video shooting the object moving with the motor command of 1.5 m/s or more. This is because the resonant mirror could not control its vibration amplitude around 0 ${}^{\circ }$ due to friction hysteresis, and there still remained small vibration as the relationship between the drive voltage and vibration amplitude was described in Section 5.1.

### 6.3. Video Shooting with Amplitude Control for Patterned Objects at Variable Speeds

**Figure 18:**
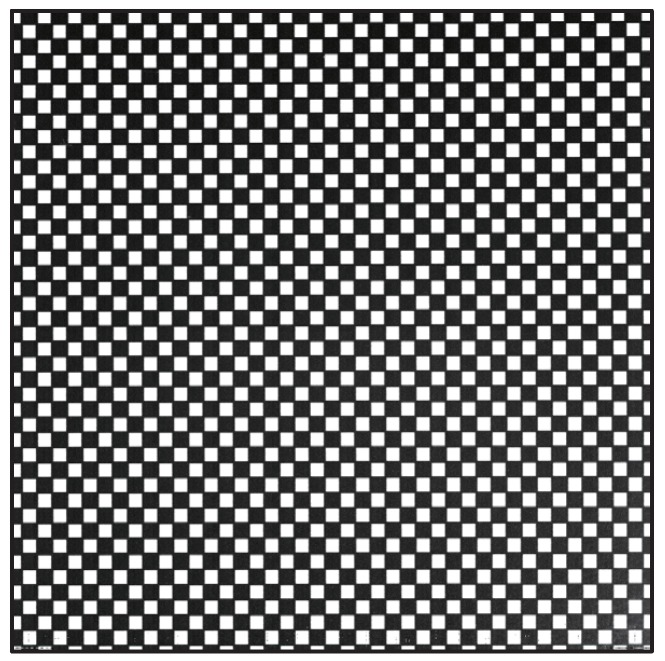
Checkered pattern to be observed.

Next, we show the experimental results in video shooting with amplitude control for a checkered pattern, when the object speed varied in the range of 0 to 7.55 m/s. In the experiments, the motor command for the belt-conveyor system was set to the following trajectory: the motor command that determines the object speed started to increase from 0 m/s at time t= 4.3 s and reached the maximum conveying speed of 7.55 m/s at time t= 8.0 s. After keeping the maximum speed during 8.0 s for t= 8.0 to 16.2 s, it started to decrease and reached 0 m/s at time t= 26.0 s. Figure 18 shows a checkered pattern on which 2 mm × 2 mm squares of alternating black and white were printed.

**Figure 19:**
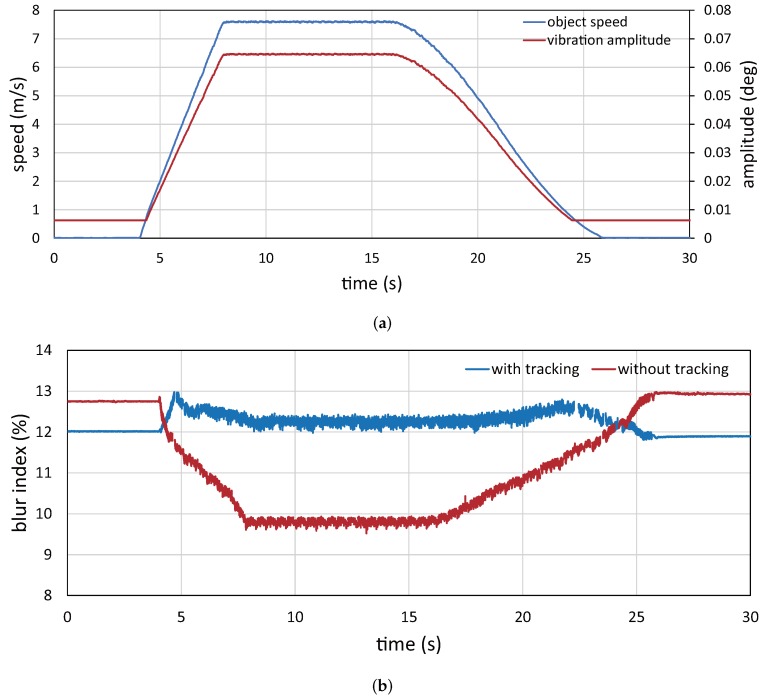
Experimental results for a checkered pattern moving at variable speeds: (**a**) object speed and vibration amplitude of resonant mirror; (**b**) blur indexes $\lambda_{edge}$ .

Figure 19 shows (a) the measured object speed and the vibration amplitude of the resonant mirror and (b) the blur index $\lambda_{edge}$ when the IT images for t= 0.0 to 30.0 s were captured with frame-by-frame intermittent tracking. The blur index $\lambda_{edge}=E_{av}/I_{ave}$ was introduced; $I_{ave}$ and $E_{ave}$ are the averaged values of the image intensities I(x,y) and edge intensities E(x,y) , respectively, for the 824 × 824 image center-cropped from a 1024 × 1024 input image. The index $\lambda_{edge}$ decreases as the motion blur becomes larger in the image, because the edge intensities in the moving direction are degraded due to motion blur. The edge intensities were computed as follows:

(13) $$\begin{array}{c} E\left(x,y\right)=\sqrt{{\left|I\left(x+1,y\right)-I\left(x,y\right)\right|}^{2}+{\left|I\left(x,y+1\right)-I\left(x,y\right)\right|}^{2}}. \end{array}$$

For comparison, Figure 19b shows the blur index $\lambda_{edge}$ when the NT images were captured with no vibration of the resonant mirror; the checkered pattern moved in a manner similar to the captured IT images. Figure 19b shows that the value of $\lambda_{edge}$ for the IT images was almost constant in the range of 11.8 to 13.0% when the speed of the checkered pattern varied in time, whereas the value of $\lambda_{edge}$ for the NT images varied considerably in the range of 9.5 to 13.0%, depending on the speed of the checkered pattern. The blur indexes $\lambda_{edge}$ for the NT images were larger than those for the IT images when the measured object speed was 0 m/s due to the non-zero offset in the vibration amplitude of the resonant mirror as illustrated in Figure 19a. There were fluctuations in the blur indexes $\lambda_{edge}$ for both the IT and NT images when the checkered pattern was moving because the average values of the edge intensities were slightly varied depending on the apparent location of the checkered pattern in the images. The dynamic response of the vibration amplitude of the resonant mirror was not so quick compared with its 750-Hz free vibration as described in Section 5.2, whereas the vibration amplitude control functioned well for motion blur reduction with frame-by-frame intermittent tracking in video shooting a target object moving at a large, but slightly time-varying speed on the belt-conveyor system, because the dynamic response of the conveyor’s speed was slower than that in the vibration amplitude control of the resonant mirror.

**Figure 20:**
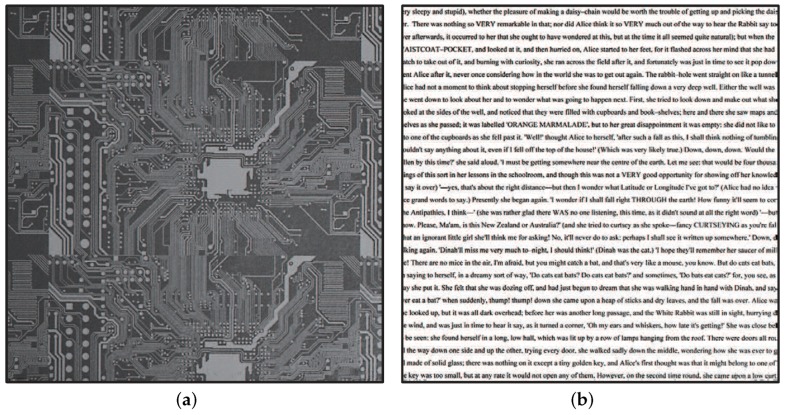
Electronic board pattern and book page pattern to be observed: (**a**) electronic board pattern; (**b**) book page pattern.

**Figure 21:**
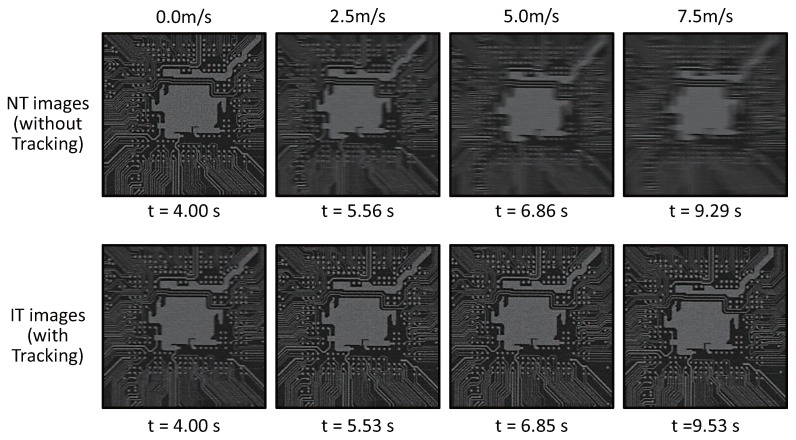
Images captured when the electronic board pattern moved at variable speeds.

**Figure 22:**
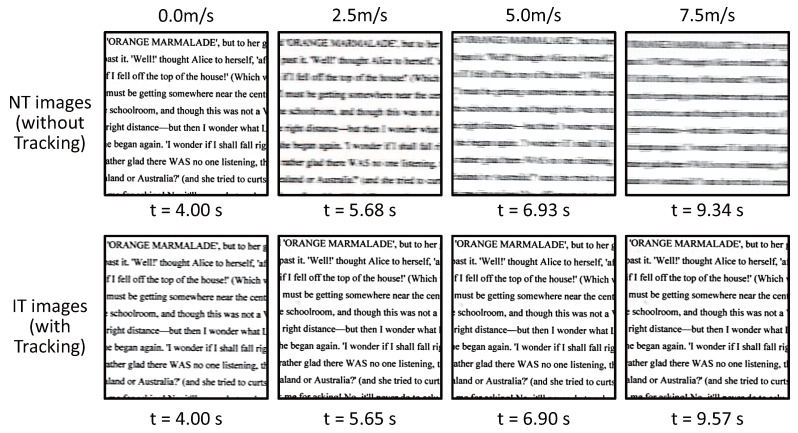
Images captured when a book page with many letters moved at variable speeds.

To verify our actuator-driven frame-by-frame intermittent tracking with amplitude control for complex patterned objects, we experimented with (a) the printed pattern of an electronic board of 54 mm × 84 mm in size with 0.25 mm-width wiring patterns and (b) the printed pattern of a book page with many 2-mm letters as illustrated in Figure 20; these patterns were attached on the belt of the belt-conveyor system and moved at variable speeds in a manner similar to the checkered pattern. Figure 21 and Figure 22 show (a) the 323 × 323 IT images cropped from the 1024 × 1024 input images of the electronic board pattern and (b) the 323 × 323 IT images of the book page pattern, when the object speed was 0.0, 2.5, 5.0 and 7.5 m/s, compared with the NT images captured when there was no vibration of the resonant mirror. The IT images at all the speeds resembled the images of unmoving patterns, which corresponded to the NT images when the object speed was 0.0 m/s, whereas the motion blur for the NT images became larger in the horizontal direction as the object speed increased. When the electronic board pattern was moving at 0.0, 2.5, 5.0 and 7.5 m/s, the blur indexes $\lambda_{edge}$ of the IT images were 11.5%, 14.8%, 15.7% and 13.4%, respectively, whereas those of the NT images were 19.8%, 9.45%, 8.13% and 7.68%, respectively. When the book page pattern was moving at 0.0, 2.5, 5.0 and 7.5 m/s, the blur indexes $\lambda_{edge}$ of the IT images were 5.22%, 7.19%, 7.59% and 6.76%, respectively, whereas those of the NT images were 8.65, 4.09, 3.62 and 3.51%, respectively. Thus, fast-moving complex patterned objects such as the wiring patterns of 0.25-mm width printed on the electronic board pattern and the 2-mm alphabet letters printed on the book page pattern were observable without noticeable blurring by applying the actuator-driven frame-by-frame intermittent tracking method with amplitude control.

## 7. Conclusions

In this study, we developed a motion-blur-free video shooting system based on the concept of actuator-driven frame-by-frame intermittent tracking. In this system, the camera frame timings are controlled for video shooting with a larger camera exposure time in synchronization with the high-frequency free vibration with a large amplitude of a resonant mirror so that it enables the ultrafast gaze control to track fast-moving objects when the camera shutter is open. Our system can capture 1024 × 1024 images of fast-moving objects at 750 fps with an exposure time of 0.33 ms without motion blur, and its performance was verified by conducting several video shooting experiments for fast-moving patterned objects on a high-speed belt-conveyor system. The following issues remain to be solved in the future. The proposed one-DOF mirror system with low-frequency response of amplitude control has a limitation in that it cannot shoot motion-blur-free videos of an object moving at a rapid time-varying speed in variable directions, and the efficacy in obtaining the incident light entering the camera is also practically limited by the size of the mirror when we use a very large aperture lens for zooming. On the basis of these experimental results and considerations, we plan to extend our motion-blur-free video shooting system to a two-DOF mirror system that can independently control the directions of the pan and tilt mirrors and improve it by feedback-controlling the camera frame timings, as well as the vibration amplitudes of resonant mirrors with high-speed real-time video processing to estimate the object speed and compute the edge-based feature for the blur index. We also plan to apply our system to various applications such as precise production inspection on a high-speed factory automation line and infrastructure inspection from a fast-moving vehicle, where video shooting with high magnification is strongly required for unidirectionally fast-moving scenes and the apparent speed of the target scene can be given as the speed of the automation line or the vehicle.

## References

[1] Kundur D., Hatzinakos D. (1996). Blind image deconvolution. IEEE Signal Proc. Mag..

[2] Campisi P., Egiazarian K. (2007). Blind Image Deconvolution: Theory and Applications.

[3] KuKim S., KiPaik J. (1998). Out-of-focus blur estimation and restoration for digital auto-focusing system. Electron. Lett..

[4] Fergus R., Singh B., Hertzbann A., Roweis S.T., Freeman W.T. (2006). Removing camera shake from a single photograph. ACM Trans. Graph..

[5] Levin A., Weiss Y., Durand F., Freeman W.T. Efficient marginal likelihood optimization in blind deconvolution. Proceedings of the IEEE Conference on Computer Vision and Pattern Recognition (CVPR).

[6] Joshi N., Szeliski R., Kriegman D.J. PSF estimation using sharp edge prediction. Proceedings of the IEEE Conference on Computer Vision and Pattern Recognition (CVPR).

[7] Cho S., Lee S. (2009). Fast motion deblurring. ACM Trans. Graph..

[8] Xu L., Jia J. Two-phase kernel estimation for robust motion deblurring. Proceedings of the European Conference on Computer Vision.

[9] Yang F., Huang Y., Luo Y., Li L., Li H. (2016). Robust image restoration for motion blur of image sensors. Sensors.

[10] Xiong N., Liu R.W., Liang M., Wu D., Liu Z., Wu H. (2017). Effective alternating direction optimization methods for sparsity-constrained blind image deblurring. Sensors.

[11] Krishnan D., Tay T., Fergus R. Blind deconvolution using a normalized sparsity measure. Proceedings of the IEEE Conference on Computer Vision and Pattern Recognition (CVPR).

[12] Joshi N., Zitnick C.L., Szeliski R., Kriegman D.J. Image deblurring and denoising using color priors. Proceedings of the IEEE Conference on Computer Vision and Pattern Recognition (CVPR).

[13] Sun L., Cho S., Wang J., Hays J. Edge-based blur kernel estimation using patch priors. Proceedings of the IEEE Conference on Computational Photography.

[14] Pan J., Sun D., Pfister H., Yang M.-H. Blind image deblurring using dark channel prior. Proceedings of the IEEE Conference on Computer Vision and Pattern Recognition (CVPR).

[15] Shan Q., Jia J., Agarwala A. (2008). High-quality motion deblurring from a single image. ACM Trans. Graph..

[16] Bascle B., Blake A., Zisserman A. Motion deblurring and super-resolution from an image sequence. Proceedings of the European Conference on Computer Vision.

[17] Chen J., Yuan L., Tang C.-K., Quan L. Robust dual motion deblurring. Proceedings of the IEEE Conference on Computer Vision and Pattern Recognition.

[18] Farsiu S., Robinson M.D., Elad M., Milanfar P. (2004). Fast and robust multiframe super resolution. IEEE Trans. Image Process..

[19] Nayar S.K., Ben-Ezra M. (2004). Motion-based motion deblurring. IEEE Trans. Pattern Anal. Mach. Intell..

[20] Tai Y.-W., Du H., Brown M.S., Lin S. Image/video deblurring using a hybrid camera. Proceedings of the IEEE Conference on Computer Vision and Pattern Recognition (CVPR).

[21] Rav-Acha A., Peleg S. (2005). Two motion-blurred images are better than one. Pattern Recognit. Lett..

[22] Yuan L., Sun J., Quan L., Shum H.-Y. (2007). Image deblurring with blurred/noisy image pairs. ACM Trans. Graph..

[23] Tai Y.-W., Tan P., Brown M.S. (2011). Richardson-Lucy deblurring for scenes under projective motion path. IEEE Trans. Pattern Anal. Mach. Intell..

[24] Whyte O., Sivic J., Zisserman A., Ponce J. (2012). Non-uniform deblurring for shaken images. Int. J. Comput. Vis..

[25] Gupta A., Joshi N., Zitnick C.L., Cohen M., Curless B. Single image deblurring using motion density functions. Proceedings of the European Conference on Computer Vision.

[26] Joshi N., Kang S.B., Zitnick C.L., Szeliski R. (2010). Image deblurring using inertial measurement sensors. ACM Trans. Graphics.

[27] Kim M.D., Ueda J. (2015). Dynamics-based motion de-blurring for a PZT-driven, compliant camera orientation mechanism. Int. J. Robot. Res..

[28] Inoue M., Jiang M., Matsumoto Y., Takaki T., Ishii I. (2017). Motion-blur-free video shooting system based on frame-by-frame intermittent tracking. ROBOMECH J..

[29] Kusaka H., Tsuchida Y., Shimohata T. (2002). Control technology for optical image stabilization. SMPTE Motion Imaging J..

[30] Cardani B. (2006). Optical image stabilization for digital cameras. IEEE Control Syst..

[31] Sato K., Ishizuka S., Nikami A., Sato M. (1993). Control techniques for optical image stabilizing system. IEEE Trans. Consum. Electron..

[32] Pournazari P., Nagamune R., Chiao M. (2014). A concept of a magnetically-actuated optical image stabilizer for mobile applications. IEEE Trans. Consum. Electron..

[33] Hao Q., Cheng X., Kang J., Jiang Y. (2015). An image stabilization optical system using deformable freeform mirrors. Sensors.

[34] Chiu C.-W., Chao P.C.-P., Wu D.-Y. (2007). Optimal design of magnetically actuated optical image stabilizer mechanism for cameras in mobile phones via genetic algorithm. IEEE Trans. Magn..

[35] Moon J.-H., Jung S.Y. (2008). Implementation of an image stabilization system for a small digital camera. IEEE Trans. Consum. Electron..

[36] Song M.-G., Hur Y.-J., Park N.-C., Park K.-S., Park Y.-P., Lim S.-C., Park J.-H. (2009). Design of a voice-coil actuator for optical image stabilization based on genetic algorithm. IEEE Trans. Magn..

[37] Song M.-G., Baek H.-W., Park N.-C., Park K.-S., Yoon T., Park Y.-P., Lim S.-C. (2010). Development of small sized actuator with compliant mechanism for optical image stabilization. IEEE Trans. Magn..

[38] Li T.-H.S., Chen C.-C., Su Y.-T. (2012). Optical image stabilizing system using fuzzy sliding-mode controller for digital cameras. IEEE Trans. Consum. Electron..

[39] Wang J.H.-S., Qiu K.-F., Chao P.C.-P. (2017). Control design and digital implementation of a fast 2-degree-of-freedom translational optical image stabilizer for image sensors in mobile camera phones. Sensors.

[40] Walrath C.D. (1984). Adaptive bearing friction compensation based on recent knowledge of dynamic friction. Automatica.

[41] Ekstrand B. (2001). Equations of motion for a two-axes gimbal system. IEEE Trans. Aerosp. Electron. Syst..

[42] Kennedy P.J., Kennedy R.L. (2003). Direct versus indirect line of sight (LOS) stabilization. IEEE Trans. Control Syst. Technol..

[43] Zhou X., Jia Y., Zhao Q., Yu R. (2016). Experimental validation of a compound control scheme for a two-axis inertially stabilized platform with multi-sensors in an unmanned helicopter-based airborne power line inspection system. Sensors.

[44] Zhang Y., Xiao Y., Zhuang Z., Zhou L., Liu F., He Y. (2016). Development of a near ground remote sensing system. Sensors.

[45] Jang S.-W., Pomplun M., Kim G.-Y., Choi H.-I. (2005). Adaptive robust estimation of affine parameters from block motion vectors. Image Vis. Comput..

[46] Xu L., Lin X. (2006). Digital image stabilization based on circular block matching. IEEE Trans. Consum. Electron.

[47] Chantara W., Mun J.-H., Shin D.-W., Ho Y.-S. (2015). Object tracking using adaptive template matching. IEIE Trans. Smart Process. Comput..

[48] Ko S.-J., Lee S.-H., Lee K.-H. (1998). Digital image stabilizing algorithms based on bit-plane matching. IEEE Trans. Consum. Electron..

[49] Ko S.-J., Lee S.-H., Jeon S.-W., Kang E.-S. (1999). Fast digital image stabilizer based on gray-coded bit-plane matching. IEEE Trans. Consum. Electron.

[50] Shen Y., Guturu P., Damarla T., Buckles B.P., Namuduri K.R. (2009). Video stabilization using principal component analysis and scale invariant feature transform in particle filter framework. IEEE Trans. Consum. Electron..

[51] Xu J., Chang H., Yang S., Wang M. (2012). Fast feature-based video stabilization without accumulative global motion estimation. IEEE Trans. Consum. Electron..

[52] Liu S., Yuan L., Tan P., Sun J. (2013). Bundled camera paths for video stabilization. ACM Trans. Graphics.

[53] Kim S.-K., Kang S.-J., Wang T.-S., Ko S.-J. (2013). Feature point classification based global motion estimation for video stabilization. IEEE Trans. Consum. Electron..

[54] Cheng X., Hao Q., Xie M. (2016). A comprehensive motion estimation technique for the improvement of EIS Methods based on the SURF algorithm and Kalman filter. Sensors.

[55] Jeon S., Yoon I., Jang J., Yang S., Kim J., Paik J. (2017). Robust video stabilization using particle keypoint update and *l*_1_-optimized camera path. Sensors.

[56] Chang J.-Y., Hu W.-F., Cheng M.-H., Chang B.-S. (2002). Digital image translational and rotational motion stabilization using optical flow technique. IEEE Trans. Consum. Electron..

[57] Matsushita Y., Ofek E., Ge W., Tang X., Shum H.-Y. (2006). Full-frame video stabilization with motion inpainting. IEEE Trans. Pattern Anal. Mach. Intell..

[58] Xu W., Lai X., Xu D., Tsoligkas N.A. (2013). An integrated new scheme for digital video stabilization. Adv. Multimed..

[59] Pathak S., Moro A., Fujii H., Yamashita A., Asama H. (2017). Spherical video stabilization by estimating rotation from dense optical flow fields. J. Robot. Mechatron.,.

[60] Edgerton H.E., Germeshausen K.J. (1934). Stroboscopic-light high-speed motion pictures. J. Soc. Motion Pict. Eng..

[61] Bradley D., Atcheson B., Ihrke I., Heidrich W. Synchronization and rolling shutter compensation for consumer video camera arrays. Proceedings of the IEEE Computer Society Conference on Computer Vision and Pattern Recognition Workshops.

[62] Boden F., Bodensiek K., Stasicki B. Application of image pattern correlation for non-intrusive deformation measurements of fast rotating objects on aircrafts. Proceedings of the Fourth International Conference on Experimental Mechanics.

[63] Theobalt C., Albrecht I., Haber J., Magnor M., Seidel H.-P. Pitching a baseball: Tracking high-speed motion with multi-exposure images. Proceedings of the ACM SIGGRAPH 2004.

[64] Borsato F.H., Aluani F.O., Morimoto C.H. A fast and accurate eye tracker using stroboscopic differential lighting. Proceedings of the IEEE International Conference on Computer Vision Workshop.

[65] Watanabe Y., Komura T., Ishikawa M. 955-fps real-time shape measurement of a moving/deforming object using high-speed vision for numerous-point analysis. Proceedings of the IEEE International Conference on Robotics and Automation.

[66] Ishii I., Taniguchi T., Sukenobe R., Yamamoto K. Development of high-speed and real-time vision platform, H3 Vision. Proceedings of the IEEE/RSJ International Conference on Intelligent Robots and Systems.

[67] Ishii I., Tatebe T., Gu Q., Moriue Y., Takaki T., Tajima K. 2000 fps Real-time vision system with high-frame-rate video recording. Proceedings of the IEEE International Conference on Robotics and Automation.

[68] Yamazaki T., Katayama H., Uehara S., Nose A., Kobayashi M., Shida S., Odahara M., Takamiya K., Hisamatsu Y., Matsumoto S. A 1ms high-Speed vision chip with 3D-stacked 140GOPS column-parallel PEs for spatio-temporal image processing. Proceedings of the IEEE International Solid-State Circuits Conference.

[69] Ishii I., Taniguchi T., Yamamoto K., Takaki T. (2012). High-frame-rate optical flow system. IEEE Trans. Circuits Syst. Video Technol..

[70] Ishii I., Tatebe T., Gu Q., Takaki T. (2012). Color-histogram-based tracking at 2000 fps. J. Electron. Imaging.

[71] Gu Q., Takaki T., Ishii I. (2013). Fast FPGA-based multiobject feature extraction. IEEE Trans. Circuits Syst. Video Technol..

[72] Gu Q., Raut S., Okumura K., Aoyama T., Takaki T., Ishii I. (2015). Real-time image mosaicing system using a high-frame-rate video sequence. J. Robot. Mechatron..

[73] Ishii I., Ichida T., Gu Q., Takaki T. (2013). 500-fps face tracking system. J. Real-Time Image Proc..

[74] Namiki A., Hashimoto K., Ishikawa M. (2003). A hierarchical control architecture for high-speed visual servoing. Int. J. Robot. Res..

[75] Senoo T., Namiki A., Ishikawa M. Ball control in high-speed batting motion using hybrid trajectory generator. Proceedings of the IEEE International Conference on Robotics and Automation.

[76] Namiki A., Ito N. Ball catching in Kendama game by estimating grasp conditions based on a high-speed vision system and tactile sensors. Proceedings of the IEEE Conference on Humanoid Robots.

[77] Aoyama T., Takaki T., Miura T., Gu Q., Ishii I. Realization of flower stick rotation using robotic arm. Proceedings of the IEEE/RSJ International Conference on Intelligent Robots and Systems.

[78] Jiang M., Aoyama T., Takaki T., Ishii I. (2016). Pixel-level and robust vibration source sensing in high-frame-rate video analysis. Sensors.

[79] Jiang M., Gu Q., Aoyama T., Takaki T., Ishii I. (2017). Real-time vibration source tracking using high-speed vision. IEEE Sens. J..

[80] Oku H., Ishii I., Ishikawa M. Tracking a protozoon using high-speed visual feedback. Proceedings of the International IEEE-EMBS Conference on Microtechnologies in Medicine and Biology.

[81] Sakuma S., Kuroda K., Tsai C.-H.D., Fukui W., Arai F., Kaneko M. (2014). Red blood cell fatigue evaluation based on the close-encountering point between extensibility and recoverability. Lab Chip.

[82] Gu Q., Aoyama T., Takaki T., Ishii I. (2015). Simultaneous vision-based shape and motion analysis of cells fast-flowing in a microchannel. IEEE Trans. Autom. Sci. Eng..

[83] Gu Q., Kawahara T., Aoyama T., Takaki T., Ishii I., Takemoto A., Sakamoto N. (2015). LOC-based high-throughput cell morphology analysis system. IEEE Trans. Autom. Sci. Eng..

[84] Yang H., Gu Q., Aoyama T., Takaki T., Ishii I. (2013). Dynamics-based stereo visual inspection using multidimensional modal analysis. IEEE Sens. J..

[85] Okumura K., Yokoyama K., Oku H., Ishikawa M. (2015). 1 ms auto pan-tilt—Video shooting technology for objects in motion based on Saccade Mirror with background subtraction. Adv. Robot..

[86] Li L., Aoyama T., Takaki T., Ishii I., Yang H., Umemoto C., Matsuda H., Chikaraishi M., Fujiwara A. Vibration distribution measurement using a high-speed multithread active vision. Proceedings of the IEEE Conference on Advanced Intelligent Mechatronics.

[87] Inoue M., Gu Q., Aoyama T., Takaki T., Ishii I. An intermittent frame-by-frame tracking camera for motion-blur-free video shooting. Proceedings of the 2015 IEEE/SICE International Symposium on System Integration.

[88] Ueno T., Gu Q., Aoyama T., Takaki T., Ishii I., Kawahara T. Motion-blur-free microscopic video shooting based on frame-by-frame intermittent tracking. Proceedings of the IEEE Conference on Automation Science and Engineering.

[89] Hayakawa T., Watanabe T., Ishikawa M. (2015). Real-time high-speed motion blur compensation system based on back-and-forth motion control of galvanometer mirror. Opt. Express.

[90] Hayakawa T., Ishikawa M. (2016). Development of motion-blur-compensated high-speed moving visual inspection vehicle for tunnels. Int. J. Civ. Struct. Eng. Res..

